# Serum Vascular Endothelial Growth Factor (VEGF-C) as a Diagnostic and Prognostic Marker in Patients with Ovarian Cancer

**DOI:** 10.1371/journal.pone.0055309

**Published:** 2013-02-01

**Authors:** Daye Cheng, Bin Liang, Yunhui Li

**Affiliations:** 1 Department of Transfusion, The First Hospital of China Medical University, Shenyang, China; 2 High Vocational Technological College, China Medical University, Shenyang, China; 3 Department of Clinical Laboratory, No.202 Hospital, Shenyang, China; Kinghorn Cancer Centre, Garvan Institute of Medical Research, Australia

## Abstract

VEGF-C is regarded as one of the most efficient factors in regulating lymphangiogenesis. The aim of this study was to better understand the role of VEGF-C in the progression of ovarian cancer and to assess its diagnostic and prognostic significance. A total of 109 patients with ovarian cancer, 76 patients with benign ovarian diseases, and 50 healthy controls were recruited in this study. Serum levels of VEGF-C were determined by ELISA method. The results showed that serum levels of VEGF-C were significantly higher in the patients with ovarian cancer than those with benign ovarian diseases and healthy controls (*P*<0.01). Serum level of VEGF-C was correlated with FIGO stage, lymph node metastasis, tumor resectability, and survival of the patients (*P*<0.05). The areas of receiver operating curves of VEGF-C were higher than those of CA125 in different screening groups. Analysis using the Kaplan-meier method indicated that patients with high VEGF-C had significantly shorter overall survival time than those with low VEGF-C (*P*<0.0001). In a multivariate analysis along with clinical prognostic parameters, serum VEGF-C was identified as an independent adverse prognostic variable for overall survival. These results indicated that serum VEGF-C may be a clinically useful indicator for diagnostic and prognostic evaluation in ovarian cancer patients.

## Introduction

Ovarian carcinoma is one of the most common cancers in women and the leading cause of death of all gynecologic tumors. In spite of recent advances, the prognosis for a woman diagnosed with advanced-stage ovarian cancer has changed little over the last thirty years with a five-year survival of only 30% [1], [2]. The relatively asymptomatic nature of early stage ovarian cancer, the rapid progression of chemoresistant disease, and the high rate of recurrence have earned ovarian cancer the reputation of being a “silent killer” [3]. Tumor invasion and metastasis are the critical steps in determining the aggressive phenotype of human cancers and the major causes of cancer deaths. Although ovarian cancer may spread in a variety of ways, dissemination to regional lymph nodes via afferent lymphatics is an important step in ovarian cancer metastasis and a major prognostic factor [4].

The vascular endothelial growth factor (VEGF) gene family, which encodes five polypeptide growth factors, VEGF-A, -B, -C, -D, and placenta like growth factor (PLGF), is particularly important because of its angiogenic and lymphangiogenic properties that promote the growth and metastasis of neoplasms. VEGF-C is regarded as the most efficient factor in regulating lymphangiogenesis [5], and it has also been shown to directly enhance the invasiveness of ovarian cancer cells in vitro [6]. To date, high expression of VEGF-C is found in breast cancer, prostatic cancer, gastric cancer, colon cancer, and lung cancer, and is associated with lymphangiogenesis, lymph node metastasis, and prognosis [5], [7]–[10].

In the progression of ovarian cancer, predictive factors can be used to gain insight into the response to a particular therapy, and prognostic factors can be useful in making decisions concerning patients who should receive adjuvant therapy. However, the diagnostic and prognostic value of serum VEGF-C in ovarian cancer is unknown. The aim of this study was to better understand the role of VEGF-C in the progression and metastasis of ovarian cancer, and to assess its prognostic significance.

## Materials and Methods

### Patients

The study included 109 consecutive ovarian cancer patients (age: 61.5±10.2 years), who were collected before surgery from the Department of Gynecology, No. 202 Hospital between July, 1999 and Sep, 2001. Clinical characteristics of the patients were given in Table 1. All patients were surgically staged in accordance with the International Federation of Gynecology and Obstetrics (FIGO) criteria. Patients who underwent preoperative chemotherapy and radiotherapy were excluded. In addition, samples of 76 age-matched patients (age: 54.1±13.5 years) with benign lesions were analyzed. Of the benign lesions, 23 were classified as mucinous cystadenomas, 7 as benign ovarian teratomas, 11 as ovarian dermoid cysts, 3 as sclerosant tumor, 8 as serous cystadenomas, 16 as fibroma, and 8 as corpus luteum cyst. A total of 50 healthy controls (age: 58.6±12.3 years) had no history of ovarian pathology or other systemic disease. Patients with ovarian cancer were monitored for survival for a median duration of 49 months (range: 1–108 months). Follow-up information was available for 106 ovarian cancer patients.

**Table 1:** Characteristics of ovarian cancer patients.

Clinicopathological parameters	Number of patients	%
Age (years)		
≥60	60	55.0
<60	49	45.0
Histology		
Serous-papillary	63	57.8
Mucinous	22	20.2
Endometroid	13	11.9
Clear cell	5	4.6
others	6	5.5
FIGO stage		
I	21	19.3
II	23	21.1
III	46	42.2
IV	19	17.4
Grading		
1	28	25.7
2	25	22.9
3	32	29.4
4	24	22.0
Residual tumor size		
≤2 cm	59	54.1
>2 cm	50	45.9
Lymph node metastasis		
No	67	61.5
Yes	42	38.5
Patients’ survival		
Survived	41	37.6
Died	65	59.6
others	3	2.8
Resectablity of tumors		
Resectable	93	85.3
Nonresectable	16	14.7

Written informed consent was obtained from all participants, and the study was approved by China Medical University Ethics Committee [2009] No.48.

### Sample Collection

Serum samples from all patients were collected presurgically, before initiation of therapy, and stored at −80°C until assessment of VEGF-C levels. Serum VEGF-C was determined with sandwich enzyme immunoassay technique (Quantikine, R & D systems, China). First, the serum samples were thawed at 4°C and then 5-fold diluted with animal serum. A total of 100µL buffered protein base and 50 µL serum sample were added to the wells of a microtiter plate coated with anti-VEGF-C mouse monoclonal antibody, and incubated for 2 hours at room temperature on a horizontal orbital microplate shaker. The process was repeated 3 times and washed 4 times. Second, 200 µL VEGF-C conjugate was added to each well, and incubated for 2 hours at room temperature on the shaker. Third, 200µL of substrate solution was added to each well, and incubate for 30 minutes at room temperature in the dark. Finally**,** color development was determined within 30 minutes at 450 nm using a microtiter plate reader after the addition of 50 µL of stop solution. Serum CA125 level was measured by electrochemiluminescence kits according to the manufacturer’s instructions and using their reagents and equipment (Roche Diagnostics, Beijing, China). All assays were performed in duplicate.

### Statistical Analysis

Statistical analyses were performed using SPSS15.0 (SPSS, Chicago, IL). The distributions of VEGF-C values in serum were asymmetric; therefore, nonparametric analyses were applied. The nonparametric Mann-Whitney sum rank U test and Kruskal-Wallis (*x* ^2^) test were used for the comparison of the variables in groups. Associations between VEGF-C and clinicopathological variables (histology, tumor stage, tumor grade, residual tumor size, lymph node metastasis, patients’ survival, and resectablity of tumors) were assessed by the chi-square test. The sensitivity and specificity of VEGF-C were determined by receiver operating characteristic (ROC) curves, and the areas under the curve were calculated [11]. The survival time was measured from the date of diagnosis to the date of death from ovarian cancer or the date of last follow-up. Using median of VEGF-C level as a cut-off, prognostic impact of VEGF-C was evaluated by the Kaplan- Meier method and the log-rank test. Furthermore, the impact of serum VEGF-C level on patient overall survival was assessed, and calculated by both univariate and multivariate Cox proportional hazards regression models. A *P* value of <0.05 was considered significant.

## Results

The levels of VEGF-C and CA125 in the sera of ovarian cancer patients and benign ovarian diseases, as well as in healthy controls were presented in Table 2. Serum levels of VEGF-C and CA125 were significantly higher in the patients with ovarian cancer than those in benign ovarian diseases and healthy controls (*P*<0.01). There were no differences between benign ovarian diseases and healthy controls for serum levels of VEGF-C and CA125 (*P*>0.05).

**Table 2:** Serum levels of VEGF-C in ovarian cancer and non-cancer groups.

	VEGF-C (pg/ml)	*P* value	CA125 (U/ml)	*P* value
Healthy controls (*n* = 50)	6338 (2459–12841)	*x^2^* = 77.47	18 (1–115)	*x* ^2^ = 72.88
Benign ovarian disease (*n* = 76)	7110 (3656–13563)	*P*<0.0001	47 (12–96)	*P*<0.0001
Ovarian cancer (*n* = 109)	10200 (4365–14563)		66 (13–522)	

Table 3 presented the levels of VEGF-C and CA125 in the sera of ovarian cancer patients in relation to clinic-pathological variables. Serum levels of VEGF-C revealed significant correlation with FIGO stage, and were the highest in the IV subgroup (*P* = 0.040). In patients with lymph node metastasis, serum levels of VEGF-C were increased in comparison with patients without lymph node metastasis (*P* = 0.010). Moreover, serum levels of VEGF-C increased in patients who died of cancer during the observation period (*P* = 0.017). Similar observation for VEGF-C was made in ovarian cancer patients with nonresectable tumors compared to those with resectable ones (*P* = 0.013).

**Table 3:** Serum levels of VEGF-C in ovarian cancer patients in relation to clinic-pathological variables of tumor.

Variables	VEGF-C (pg/ml)	*P* value	CA125(U/ml)	*P* value
Histology				
Serous-papillary	9633(4365–14563)	*x* ^2^ = 8.763	69(13–522)	*x* ^2^ = 7.380
Mucinous	9743(6855–13500)	*P* = 0.067	36(13–366)	*P* = 0.117
Endometroid	10850(8520–13285)		76(23–510)	
Clear cell	12525(11000–13200)		66(36–155)	
others	11874(9585–12354)		98(66–121)	
FIGO stage				
I–II	9693(4365–14255)	*x* ^2^ = 8.339	36(13–366)	*x* ^2^ = 4.299
III–IV	11582(9241–13285)	*P = *0.040	96(33–222)	*P* = 0.231
Grading				
1–2	10297(4365–14563)	*x* ^2^ = 5.735	37(13–522)	*x* ^2^ = 2.545
3–4	9555(6258–11414)	*P* = 0.057	85(23–512)	*P* = 0.282
Residual tumor Size				
≤2 cm	9633(4365–14563)	*x* ^2^ = 2.931	66(13–522)	*x* ^2^ = 0.038
>2 cm	11028(6855–13500)	*P* = 0.087	68(16–510)	*P* = 0.846
Lymph node metastasis				
No	9633(4365–14563)	*x* ^2^ = 6.693	66(13–522)	*x* ^2^ = 0.221
Yes	11000(6855–13500)	*P* = 0.010	71(16–510)	*P = *0.638
Patients’ survival				
Survived	9612(4365–14563)	*x* ^2^ = 5.748	60(13–522)	*x* ^2^ = 1.128
Died	10925(6855–13500)	*P* = 0.017	65(16–510)	*P = *0.288
Resectablity of tumors				
Resectable	9744(4365–14563)	*x* ^2^ = 6.180	64(13–522)	*x* ^2^ = 0.001
Nonresectable	11874(9241–13285)	*P* = 0.013	98(33–222)	*P = *0.970

**Figure 1:**
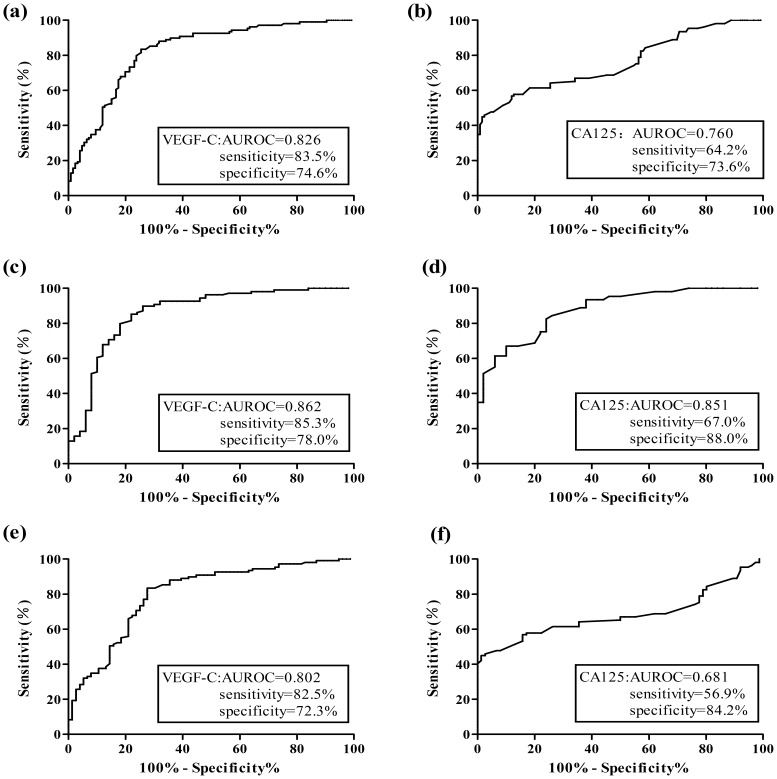
Receiver-operating curve (ROC) analysis of VEGF-C and CA125 in the detection of ovarian cancer. (a–b) ROC analysis included patients with benign ovarian diseases and healthy controls as a negative group and patients with ovarian cancer as a positive group. (c–d) ovarian cancer patients *vs* healthy controls; (e–f) ovarian cancer patients *vs* benign ovarian diseases. AUROC: area under receiver-operating curve.

As shown in Figure 1(a), area under receiver operating curve (AUROC) analysis assessing serum VEGF-C as a diagnostic tool for discriminating ovarian cancer from benign ovarian diseases and healthy controls was 0.826 (95% CI, 0.773–0.879) compared with 0.760 (95% CI, 0.697–0.822) for CA125. Additionally, Figure 1(b) showed that AUROC of VEGF-C for differentiating ovarian cancer from health controls was 0.862 (95% CI, 0.794–0.931), which was higher than 0.853 (95% CI, 0.773–0.933) of CA125. Figure 1(c) presented that the approximate AUROC for ovarian cancer versus benign ovarian diseases was 0.802 (95% CI, 0.736–0.868) and 0.681 (95% CI, 0.604–0.758) for VEGF-C and CA125, respectively. AUROC of VEGF-C was larger than CA125 in different screening groups.

**Figure 2:**
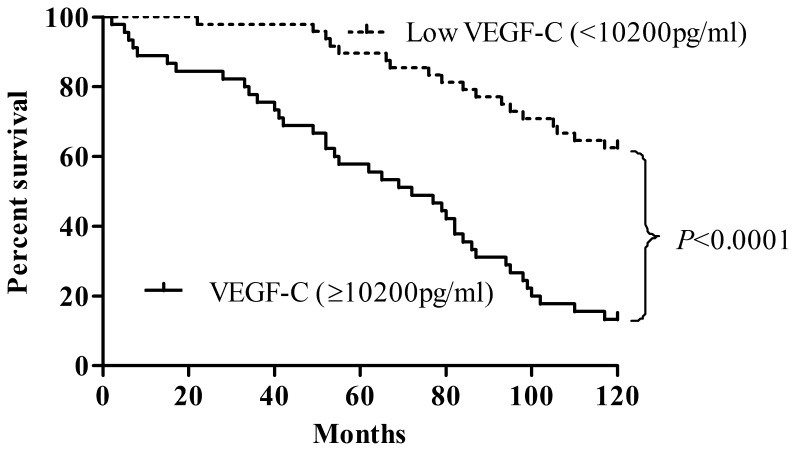
Kaplan-Meier survival curves. Percent survival rate was stratified by VEGF-C level.

Analysis using Kaplan-Meier method showed that patients with high level of VEGF-C (≥10200 pg/ml) had significantly shorter overall survival than those with low level of VEGF-C (<10200 pg/ml), as shown in Figure 2 (*P*<0.0001). The curve indicated that high level of VEGF-C is significantly associated with an increased risk of death. To evaluate factors that affected overall survival, the five clinical factors and VEGF-C level listed in Table 4 were included in the analysis. Univariate analysis revealed that tumor stage (III and IV) (*P*<0.0001), lymph node metastasis (*P*<0.001), and high levels of VEGF-C (*P* = 0.001) were significantly associated with survival of patients with ovarian cancer. Of these, tumor stage (III and IV) (*P* = 0.006), lymph node metastasis (*P* = 0.021), and high levels of VEGF-C (*P* = 0.01) remained significant on multivariate analysis. Among the factors studied, VEGF-C level was an independent predictor of poor patient survival.

**Table 4:** Univariate and multivariate survival analysis in patients with ovarian cancer.

	Overall survival		
	Univariate	Multivariate	
	*P* value	HR (95% CI)	*P* value
Tumor stage (I,II vs. III,IV)	<0.0001	1.301–4.784	0.006
Histopathology (Serous-papillary vs. others)	0.325		
Grading (1,2 vs.3,4)	0.212		
Residual tumor(≤2 cm vs. >2 cm)	0.554		
Lymph node metastasis (positive vs. negative)	<0.0001	1.126–4.189	0.021
Serum VEGF-C (≥10200 pg/ml vs. <10200 pg/ml)	0.001	1.256–5.501	0.01

## Discussion

The metastatic spread of tumor cells is responsible for the majority of cancer deaths. Lymphangiogenesis and sustained angiogenesis are important steps in tumor progression. Similar to angiogenesis, a tumor can induce its own network of lymphatics that connect with the surrounding lymphatic vessels. To date, clinical and pathological results indicate that the metastasis of tumor cells by lymphatics is the most common pathway of initial dissemination for many carcinomas. Lymphangiogenesis was reported to play a role in ovarian cancer progression both clinically and in experimental models [12], [13].

As a member of VEGF family, VEGF-C is first demonstrated to induce lymphangiogenesis and promote metastasis in animal studies, and is also expressed in a variety of human adult tissues including heart, placenta, muscle, ovary, and small intestine [14], [15]. Previous studies have demonstrated that VEGF-C/VEGFR3 signaling system is regarded as the most efficient pathway in regulating lymphangiogenesis. VEGF-C secreted by tumor cells can specifically act on receptor VEGFR-3 at the surface of lymphatic endothelial cells, thus activating the signaling system for tumor lymphangiogenesis. Clinicopathological correlations have shown that there is a strong correlation between VEGF-C/VEGFR3 signaling and lymph node metastasis in various human cancers [16], [17]. Nakazato T, *et al*. reported that VEGF-C expression in oral squamous cell carcinoma triggers lymphangiogenesis, which may result in a high risk for cervical lymph node metastasis [18]. Schietroma C *et al*, demonstrated that VEGF-C positive lymphatic density is increased around malignant melanoma and associated with tumor cells invasion of the lymph node [19]. Recent studies identified VEGF-C as a prognostic factors for poor prognosis in gastric adenocarcinoma [20], cholangiocarcinoma [21], breast cancer [22], and lung cancer [23].

Neoplastic angiogenesis and lymphangiogenesis are essential for the growth of tumor tissue in both primary and metastatic sites. This implies that the detection of angiogenic and lymphangiogenic factors may indicate the presence of malignant tumor at an early stage. Data focusing on lymphangiogenesis in ovarian cancer are scant. However, Sinn BV, *et al*, demonstrated that VEGF-C mRNA is associated with aggressive tumor behavior in ovarian cancer [24]. Although, an increase in VEGF-C mRNA was observed, the relationship between serum VEGF-C levels and tumor behavior has not yet been determined by a quantitative method in vivo. In our study, we analyzed serum VEGF-C levels in a large number of ovarian cancer samples using ELISA method and focusing on different subtypes, serous-papillary adenocarcinoma, mucinous adenocarcinoma, endometroid tumor, clear cell ovarian tumor and others, which differ substantially in a number of genetic, biological, pathological and clinical features. The results showed that the levels of VEGF-C were elevated in ovarian cancer patients compared with benign ovarian diseases and healthy controls. Therefore, in order to further evaluate the clinical value of VEGF-C, we determined and compared the diagnostic usefulness of VEGF-C and CA125 in different screening groups. Serum VEGF-C levels distinguished ovarian cancer patients from normal individuals and/or benign ovarian disease patients very reliably. Serum VEGF-C was a significantly better predictor for ovarian cancer than CA125 in this studied population.

The levels of VEGF-C in the sera of 109 ovarian cancer patients were assessed in relation to clinicopathological features of tumor such as FIGO stage, grading, tumor location, lymph node metastasis, tumor resectability, and survival of the patients. Additionally, serum VEGF-C increased significantly with FIOGO stage, lymph node metastasis, survival of patients, and tumor resectablility. Our results are in agreement with the findings of Huang KJ, *et al* [25], who revealed significant difference of VEGF-C in lymph node metastasis. As described above, it suggests that the elevated levels of VEGF-C might reflect the role of this protein as a stimulator of lymphangiogenesis and tumor cell metastasis in ovarian cancer progression.

Finally, survival analysis of the ovarian cancer patients was evaluated in this study based on the levels of VEGF-C. A previous study has illustrated the potential utility of VEGF-C as a prognostic marker in breast cancer patients [26]. In our study, survival rate of patients with high VEGF-C level was significantly lower than the patients with low VEGF-C level (*P*<0.0001). Of note, preoperative levels of CA125 in these patients did not correlate with a poor outcome (data not shown). Besides the already established prognostic predictor, such as FIGO stage and lymph node metastasis, serum VEGF-C was independently associated with prognosis in all patients with ovarian cancer. Thus, serum VEGF-C can identify early-stage patients who are of disease-related death. This could well influence treatment decisions.

Our results suggested that VEGF-C may be involved in the progression of ovarian cancer, and that high level of VEGF-C in sera may be related to lymphangiogenesis of the tumor as well as the invasion of the lymphatic vessels in ovarian cancer. As a noninvasive marker, VEGF-C has the potential to clinically expedite the diagnosis of ovarian cancer and to aid in the detection of lymph node metastasis in women for whom CA125 is not useful. Overall, the addition of VEGF-C to the diagnostic tool in cases with suspected widespread ovarian cancer might contribute to the accuracy of the diagnosis, sparing intensive endoscopic search for disseminated foci. It could also be used for the follow-up of those patients, and predict the outcome of patients.

Our finding has several limitations. First, validation of the predictive significance of VEGF-C requires large-scale studies on homogenous populations. It is unlikely that clinicopathological subgroups of adequate size could be pooled within a single institution. Second, VEGF-C is not specific for ovarian cancer and they are implicated in both tumor- and inflammation-induced lymphangiogenesis. Third, regarding the ability of VEGF-C to discriminate between early ovarian cancer and benign diseases, less common histological types, and cases where CA125 is low, we need recruit more clinical cases and make an analysis of difference in depth. Finally, the optimal methodology for the evaluation of serum VEGF-C has not yet been standardized and the “normal” levels are unknown. Further validations are needed in large studies to better investigate the clinical implications of serum VEGF-C level and to determine if serum VEGF-C can provide more useful information regarding the presence of early stage ovarian cancer, distant metastasis, and prognostic value of ovarian cancer.
